# A Variação Anormal da Pressão Arterial Circadiana está Associada aos Escores SYNTAX em Pacientes Hospitalizados com Síndrome Coronariana Aguda

**DOI:** 10.36660/abc.20210546

**Published:** 2022-04-26

**Authors:** Turhan Turan, Ahmet Özderya, Sinan Sahin, Selim Kul, Ali Hakan Konuş, Faruk Kara, Gulay Uzun, Ali Rıza Akyüz, Muhammet Rasit Sayin

**Affiliations:** 1 Trabzon Ahi Evren Thoracic and Cardiovascular Surgery Training and Research Hospital University of Health Sciences Trabzon Turquia Trabzon Ahi Evren Thoracic and Cardiovascular Surgery Training and Research Hospital - University of Health Sciences, Trabzon – Turquia

**Keywords:** Hipertensão, Monitorização Ambulatorial da Pressão Arterial, Síndrome Coronariana Aguda, Pacientes Internados, Doença da Artéria Coronariana

## Abstract

**Fundamento:**

Menor redução da pressão arterial (PA) noturna, conhecida como hipertensão não-dipper, é um forte preditor de morbimortalidade cardiovascular.

**Objetivos:**

Este estudo visou investigar a relação entre a hipertensão não-dipper e a gravidade e complexidade da doença arterial coronariana usando o escore SYNTAX em pacientes hospitalizados com síndrome coronariana aguda.

**Métodos:**

Foram selecionados 306 pacientes consecutivos com síndrome coronariana aguda. Pacientes clinicamente estáveis internados na unidade de terapia intensiva intermediária pelo menos 24 horas após a angiografia e/ou revascularização bem sucedida. Após os critérios de exclusão, foram incluídos 141 pacientes (34 mulheres e 107 homens; idade média 61 ± 11 anos). A hipertensão não-dipper foi definida como uma queda de 0% a 10% na PA sistólica média durante a noite em comparação com o dia, medida em intervalos de 1 hora, usando o mesmo dispositivo automático de medição de PA em monitores de beira de leito (Vismo PVM-2701; Nihon Kohden Corp., Tóquio, Japão). O escore SYNTAX foi calculado com uma calculadora online. Os preditores independentes do escore SYNTAX foram avaliados por meio de análise de regressão logística multivariada. P < 0,05 foi considerado estatisticamente significativo.

**Resultados:**

Os pacientes com hipertensão não-dipper apresentaram escore SYNTAX maior do que os pacientes com hipertensão dipper (11,12 ± 6,41 versus 6,74 ± 6,45, p < 0,0001). Em um modelo de regressão logística multivariável, o status de hipertensão não dipper (odds ratio: 5,159; intervalo de confiança de 95%: 2,246 a 11,852, p < 0,001), sexo (p = 0,012) e colesterol de lipoproteína de baixa densidade (p = 0,008) emergiram como preditores independentes de alto escore SYNTAX.

**Conclusões:**

Os resultados do nosso estudo fornecem um possível mecanismo adicional ligando o perfil anormal da PA circadiana à gravidade e à complexidade da doença arterial coronariana em pacientes com síndrome coronariana aguda.

## Introdução

A pressão arterial (PA) fisiológica apresenta um padrão circadiano com queda de 10% a 20% durante o sono em relação à PA diurna. Essa diminuição durante o sono é definida como dipping extremo quando ≥ 20%, dipping normal quando encontra-se entre 10% e 20%, não-dipping quando < 10% e dipping reverso se houver algum aumento (relação noite-dia: ≤ 0,8, < 0,8 a ≤ 0,9, < 0,9 a ≤ 1, e > 1, respectivamente).^1^ Menor redução da PA noturna é um forte preditor de morbimortalidade cardiovascular tanto para pacientes com hipertensão quanto para pacientes sem.^2-7^ O método padrão para determinar os padrões não-dipper e dipper em pacientes é a monitorização ambulatorial da pressão arterial (MAPA) não invasiva de 24 horas, que é geralmente realizada fora do consultório. Por outro lado, alternativamente, a monitorização clínica da pressão arterial (MCPA) em pacientes hospitalizados e a monitorização domiciliar da pressão arterial (MDPA) em pacientes ambulatoriais são realizadas com medidas manuais ou medidas automáticas infrequentes da PA. A MCPA e a MRPA mostraram anteriormente terem medidas de PA diurna e noturna semelhantes à MAPA em pacientes hospitalizados e ambulatoriais e serem consistentes com a MAPA na verificação de hipertensão não-dipper.^8-10^

Embora os distúrbios diurnos da PA estejam ligados a danos em diversos órgãos e eventos cardiovasculares, o mecanismo subjacente não é claro.^11-13^ No entanto, o significado clínico da variação circadiana anormal da PA em pacientes internados no hospital com um evento cardiovascular recente ainda não foi estudado. O escore do estudo Synergy between Percutaneous Coronary Intervention with Taxus and Cardiac Surgery (SYNTAX) é um dos sistemas de escore angiográfico coronariano detalhado mais aceito para determinar a gravidade e a complexidade da doença arterial coronariana (DAC), dependendo da anatomia coronariana e características da lesão.^14-16^ O presente estudo visou avaliar a relação entre o escore SYNTAX e menor dipping noturno da PA com uso frequente de MCPA (em intervalos de 1 hora) em pacientes hospitalizados com síndrome coronariana aguda (SCA).

## Métodos

### População do estudo

**Figura 1:**
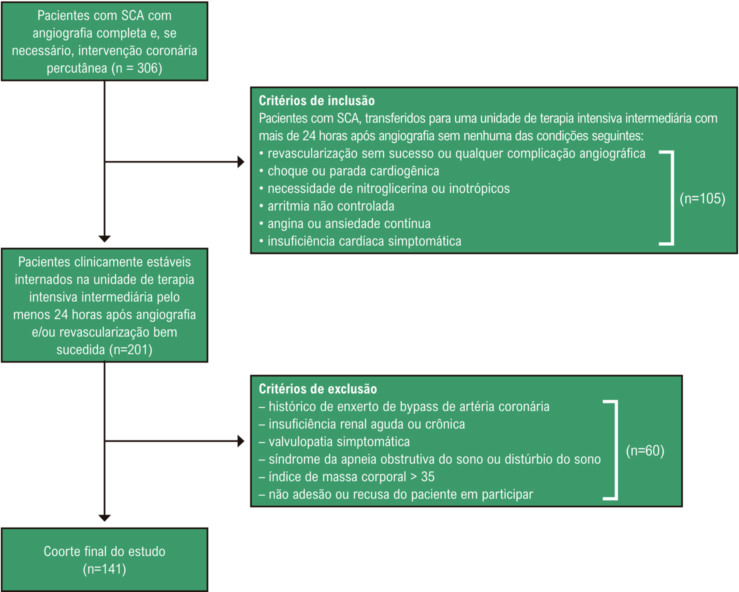
– Fluxograma do estudo. SCA: síndrome coronariana aguda.

O presente estudo prospectivo, transversal e de centro único foi realizado entre janeiro e abril de 2020 no Centro Torácico e Cardiovascular Ahi Evren, em Trabzon, Turquia. Os participantes do estudo foram recrutados prospectivamente de um total de 306 pacientes com SCA (infarto do miocárdio com supradesnivelamento do segmento ST [IAMCSST], infarto agudo do miocárdio sem supradesnivelamento do segmento ST [IAMSSST], angina pectoris instável), que haviam sido submetidos à angiografia coronária. Foram aferidos parâmetros bioquímicos, incluindo colesterol total, lipoproteína de baixa densidade (LDL), lipoproteína de alta densidade (HDL), triglicerídeos e testes de função renal. Foram medidos os parâmetros hematológicos como parte do hemograma completo automatizado (analisador automático de hematologia Mindray BC-5800, Mindray Medical Electronics Co. Shenzhen, China). A hipertensão foi diagnosticada e estratificada de acordo com as diretrizes recentes.^17^ Os pacientes que haviam recebido tratamento anti-hipertensivo anteriormente continuaram os mesmos tratamentos durante todo o período de acompanhamento. Demos medicamentos anti-hipertensivos a todos os pacientes nos horários da manhã sem alterar o seu uso. A hipercolesterolemia foi definida como colesterol total > 200 mg/dl. A taxa de filtração glomerular estimada foi calculada usando a fórmula de Cockcroft-Gault.^18^ Excluímos pacientes com qualquer uma das condições a seguir: apresentando choque ou parada cardiogênica, recebendo nitroglicerina intravenosa ou terapia inotrópica por qualquer motivo, histórico de enxerto de bypass de artéria coronária, valvulopatias, malignidade, doença renal ou hepática, insuficiência cardíaca sintomática, hipertensão secundária, arritmia não controlada, angina ou ansiedade contínua, síndrome da apneia obstrutiva do sono ou distúrbio do sono e obesidade mórbida (índice de massa corporal > 35). Por fim, a população do estudo consistiu em 141 pacientes clinicamente estáveis, incluindo 85 com IAMSSST, 15 com angina pectoris instável e 41 com IAMCSST (Figura 1). A idade dos pacientes variou de 32 a 91 anos. O protocolo do estudo estava em conformidade com os princípios da Declaração de Helsinki e recebeu aprovação do Comitê de Ética Institucional local. O consentimento informado foi obtido de cada participante do estudo.

### Angiografia coronária

Todos os pacientes foram submetidos à angiografia coronária em 24 horas. O tempo médio dos sintomas até a angiografia coronária foi de aproximadamente 2 a 6 horas. Foi realizada a angiografia coronária pela técnica padrão de Judkins utilizando cateteres de 6 ou 7 Fr (Expo, Boston Scientific Corporation, Massachusetts, EUA) através da artéria femoral. Quando necessária, a intervenção coronária percutânea para a lesão culpada foi realizada com sucesso em pacientes elegíveis na mesma sessão (120/141 pacientes, 85%). O escore SYNTAX foi calculado usando uma calculadora online conforme descrito na literatura de acordo com os achados angiográficos basais por 2 operadores experientes que desconheciam os demais parâmetros.^16^

### Medição da pressão arterial e protocolo de estudo

Na nossa clínica, os pacientes com SCA são acompanhados na unidade de terapia intensiva pelo menos nas primeiras 24 horas após a intervenção coronária percutânea. No entanto, pacientes estáveis de baixo risco (pacientes com revascularização bem-sucedida, sem arritmia maligna, dor aliviada e sem sinais de insuficiência cardíaca) são mobilizados e acompanhados na unidade de terapia intensiva intermediária ao final de 24 horas. A nossa população de estudo foi selecionada a partir desses pacientes e foram realizadas medidas de PA de hora em hora na unidade de terapia intensiva intermediária com um dispositivo automático de medição de PA em monitores de beira de leito para todos os pacientes (Vismo PVM-2701, Nihon Kohden Corp., Tóquio, Japão). As medidas foram feitas no membro superior usando 2 manguitos infláveis (22 × 12 e 30 × 14 cm) para cobrir pelo menos 80% da circunferência do braço do paciente. Foi garantida a acurácia da medição otimizando todos os monitores dos pacientes antes das primeiras medições e comparando-os com medições de esfigmomanômetros de padrão calibrados. Ao permitir que os pacientes dormissem em suas camas às 23:00 h e acordassem às 7:00 h, os valores noturnos da PA foram obtidos por medição a cada hora. Informamos a todos os pacientes antes do procedimento e realizamos todas as medidas da PA noturna enquanto os pacientes dormiam.

Excluímos a medida se o paciente acordasse por qualquer motivo, e a medida foi repetida imediatamente após o paciente dormir. Beber café, fumar e fazer exercícios não foram permitidos antes da medição; após sentar e descansar por 5 minutos, a PA diurna foi obtida pela medida da PA a cada hora entre 08:00 h e 22:00 h com o mesmo aparelho, em decúbito dorsal. Para todas as medições, os monitores dos pacientes foram configurados para medir em intervalos de 1 hora. O mesmo profissional de saúde experiente verificou os pacientes em termos de estado de sono-vigília, adequação do manguito e posição do paciente durante a medição; as medidas noturnas da PA foram registradas sob luz fraca, sem acender totalmente as luzes da unidade de terapia intensiva intermediária. Caso o paciente fosse transferido para a unidade de terapia intensiva intermediária em horário que não correspondesse ao início dos períodos de medição, iniciamos as medições no primeiro período seguinte (23:00 da noite ou 7:00 do dia). Os pacientes foram acompanhados na unidade de terapia intensiva intermediária durante pelo menos 24 horas. Valores extremos de PA (PA sistólica > 200 mmHg ou < 90 mmHg; PA diastólica > 110 mmHg ou < 40 mmHg) foram considerados errôneos e não foram incluídos na análise. Considerando as médias dos valores horários de PA para 9 períodos noturnos (23:00 h às 07:00 h) e 15 períodos diurnos (8:00 h às 22:00 h), obteve-se um único valor médio de PA diurna e noturna. A queda da PA sistólica noturna para diurna foi calculada como 100 × ([média da PA sistólica diurna – média da PA sistólica noturna]/média da PA sistólica diurna]). A redução da PA durante a noite em relação ao dia foi definida da maneira seguinte: dipping normal quando estava entre 10% e 20%, não-dipping quando < 10% e dipping reverso quando houve algum aumento (relação noite-dia: ≤ 0,8, < 0,8 a ≤ 0,9, < 0,9 a ≤ 1, e > 1, respectivamente).

### Avaliação ecocardiográfica

Foi realizado o exame ecocardiográfico com um sistema de ultrassom cardiovascular disponível comercialmente (Vivid 5, GE Vingmed, Horten, Noruega). Foi realizada a aquisição de dados com um transdutor de 1,5 a 2,6 MHz nas cortes paraesternal e apical (visão padrão de 2 e 4 câmaras). Foram obtidas imagens bidimensionais e Doppler enquanto os pacientes estavam prendendo a respiração e armazenadas em formato cine-loop a partir de 3 batimentos consecutivos; valores médios foram relatados e os eletrocardiogramas foram registrados simultaneamente. A fração de ejeção do ventrículo esquerdo foi derivada pela regra de Simpson modificada biplano e apical. As medidas do Doppler incluíram a velocidade de pico de enchimento mitral precoce (onda E), a velocidade de pico de enchimento mitral tardia (onda A) e a relação entre as velocidades de pico de enchimento mitral precoce e tardia (E/A). Para as velocidades do tecido miocárdico, o volume da amostra de imagem do Doppler tecidual foi colocado no anel mitral lateral na junção entre o corte da parede lateral do ventrículo esquerdo e o corte apical de 4 câmaras do anel mitral. A imagem Doppler tecidual incluiu os seguintes parâmetros: velocidade miocárdica diastólica precoce (Em), velocidade miocárdica diastólica tardia (Am) e Em/Am. Todos os ecocardiogramas foram interpretados por 2 cardiologistas experientes cegos ao estado do paciente.

### Análise estatística

O SPSS 21.0 for Windows (SPSS Inc., Chicago, IL, EUA) foi utilizado para as análises estatísticas. Os dados quantitativos foram expressos como média ± desvio padrão. Os dados categóricos foram apresentados como número e frequência (%). Para a técnica de análise adequada, foram aplicados os testes de Kolmogorov–Smirnov e de homogeneidade de variância. Foram usados testes t de amostras independentes para comparação de 2 grupos das variáveis normalmente distribuídas e foi usado o teste U de Mann-Whitney para comparação de 2 grupos das variáveis sem distribuição normal. As variáveis com distribuição não normal foram expressas como medianas (intervalos interquartis). As variáveis contínuas normalmente distribuídas foram expressas como média ± desvio padrão. As variáveis categóricas foram comparadas pelo teste de qui-quadrado. Os preditores independentes do escore SYNTAX foram avaliados por meio de análise de regressão logística multivariada. No procedimento multivariado, idade, sexo, índice de massa corporal, histórico de hipertensão e diabetes mellitus, tabagismo, taxa de filtração glomerular, níveis de colesterol LDL e hipertensão não-dipper foram as variáveis clínicas consideradas. P < 0,05 foi considerado estatisticamente significativo.

## Resultados

**Figura 2:**
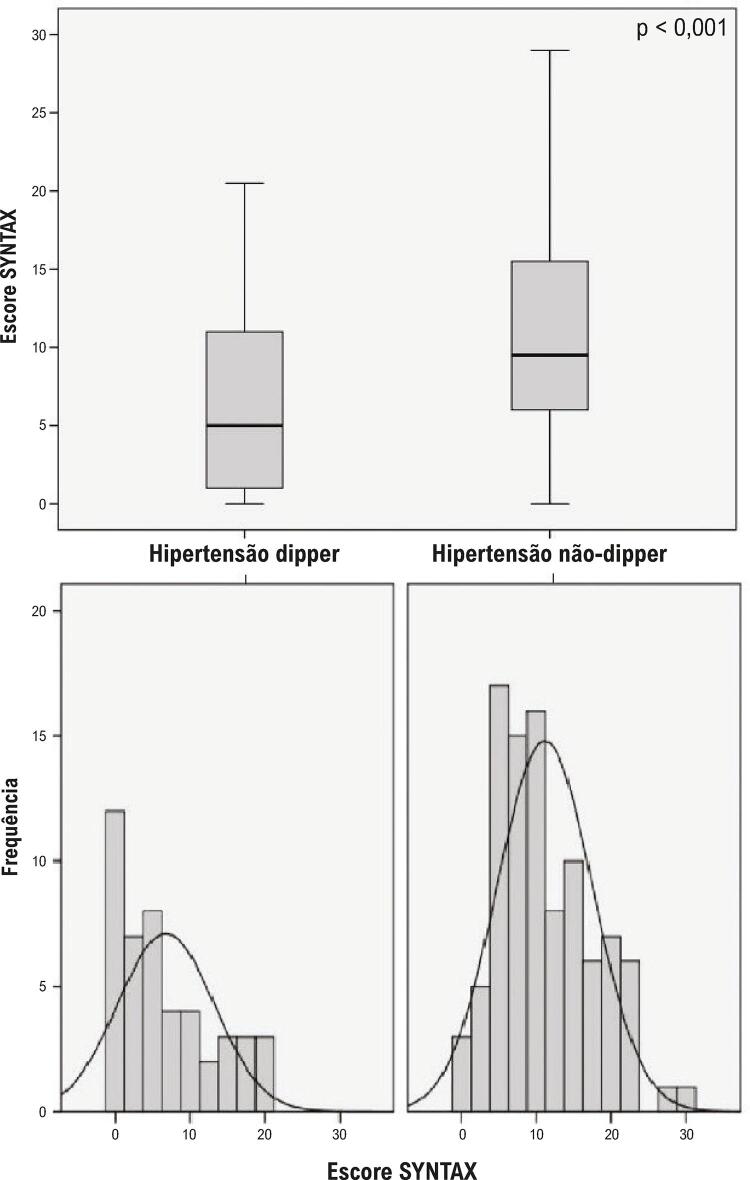
– Comparação dos escores SYNTAX de pacientes nos grupos de hipertensão dipper e não-dipper (11,12 ± 6,41 versus 6,74 ± 6,45, p < 0,001).

Neste estudo, foram selecionados 306 pacientes consecutivos com SCA. Após a exclusão dos pacientes que preencheram os critérios de exclusão, os 141 pacientes restantes (34 do sexo feminino e 107 do sexo masculino; idade média de 61 ± 11 anos) foram incluídos no estudo (Figura 1). IAMCSST, IAMSSST e angina pectoris instável foram observados em 41 (29%), 85 (60%) e 15 (11%) pacientes, respectivamente. Entre todos os pacientes com SCA, a hipertensão não-dipper foi observada em 95 (67%) pacientes. As características clínicas dos pacientes são apresentadas na Tabela 1. Houve diferenças clínicas significativas e notáveis entre os grupos. Os pacientes com hipertensão não-dipper apresentaram maior porcentagem de IAMCSST e menor porcentagem de angina pectoris instável do que pacientes com hipertensão dipper. Os pacientes com hipertensão não-dipper também apresentaram escore SYNTAX mais alto, níveis mais altos de pico de troponina I de alta sensibilidade, dimensões ventriculares esquerdas mais altas e fração de ejeção mais baixa do que os pacientes com hipertensão dipper (Tabelas 1 e 2 e Figura 2).

**Tabela 1:** – Características clínicas basais da população do estudo

	Grupo dipper (n=46)	Grupo não-dipper (n=95)	p
Idade (anos)	61±12	61±11	0,868^a^
Sexo masculino (n) (%)	35 (76)	72 (76)	0,969^a^
IMC (kg/m^2^)	27,4±3,8	28,0±3,7	0,395^a^
HT (n, %)	23 (50)	48 (51)	0,953^b^
DM (n, %)	12 (26)	29 (31)	0,586^b^
Tabagismo (n, %)	14 (30)	36 (38)	0,385^b^
PA diurna média (mmHg, sistólica/diastólica)	121,3±13,9/ 73,4±9,2	118,2±15,4/ 69,5±10,3	0,251^a^ 0,035^a^
PA noturna média (mmHg, sistólica/diastólica)	103,5±12,3/ 63,0±8,2	118,2±15,4/ 70,5±9,8	<0,001^a^ <0,001^a^
Tipo de SCA			
IAMCSST (n, %)	7 (15)	34 (36)	0,012^b^
IAMSSST (n, %)	29 (63)	56 (59)	0,641^b^
API (n, %)	10 (22)	5 (5)	0,030^b^
Escore SYNTAX*	5 (0-21)	9,5 (0-29)	<0,001^c^
Escore SYNTAX alto (n, %)^ #^	16 (35)	64 (67)	<0,001^b^
Medicações			
Inibidor da ECA ou BRA (n, %)	16 (35)	30 (32)	0,704^b^
Antagonista de cálcio (n, %)	6 (13)	21 (22)	0,200^b^
Diurético (n, %)	8 (17)	17 (18)	0,942^b^
ARM (n, %)	1 (2)	2 (2)	0,979^b^
Β-bloqueador (n, %)	10 (22)	27 (28)	0,398^b^
α-bloqueador (n, %)	1 (2)	0 (0)	0,149^b^
Clopidogrel	35(76,1)	84(88)	0,082^ b^
Ticagrelol	8 (17)	9 (9,5)	0,180^ b^
Prasugrel	3 (6,5)	2(2,1)	0,330^ b^
Ácido acetilsalicílico	46 (100)	95 (100)	1^ b^
Estatina	46 (100)	93 (98)	1^ b^

^a ^Teste t independente, ^b ^Teste do qui-quadrado, ^c ^Teste U de Mann–Whitney, * Os dados são expressos como mediana (intervalo interquartil) para as variáveis contínuas. ^#^ Acima do valor mediano. API: angina pectoris instável; ARM: antagonista do receptor mineralocorticoide; BRA: bloqueador do receptor de angiotensina; DM: diabetes mellitus; ECA: enzima conversora de angiotensina; HT: hipertensão; IAMCSST: infarto do miocárdio com supradesnivelamento do segmento ST; IAMSSST: infarto do miocárdio sem supradesnivelamento do segmento ST; IMC: índice de massa corporal; PA: pressão arterial; SCA: síndrome coronariana aguda.

**Tabela 2:** – Valores bioquímicos e parâmetros ecocardiográficos da população do estudo

	Grupo dipper n=46	Grupo não-dipper n=95	p
Pico de troponina I de alta sensibilidade (ng/L) *	2413 (13,9-50000)	9036 (1,01-150050)	0,021^c^
Creatinina (mg/dL) *	0,94 (0,56-1,66)	0,87 (0,63-1,86)	0,361^c^
eTFG (ml/dk/1,73 m^2^) *	83,5 (34-114)	89,6 (32,9-118)	0,737^c^
Sódio (mmol/L)	137±2	137±2	0,549^a^
Potássio (mmol/L)	4,3±0,3	4,3±0,4	0,930^a^
Cálcio (mg/dL)	9,0±0,4	8,8±0,5	0,058^a^
ALT (IU/L) *	24 (9-94)	24 (6-150)	0,418^c^
AST (IU/L) *	35,5 (15-291)	38 (10-472)	0,809^c^
PCR (mg/L) *	2,65 (0-79)	2,4 (0-93)	0,974^c^
Glicemia de jejum (mg/dL) *	122,5 (74-367)	127 (59-316)	0,427^c^
LDL-C (mg/dL)	137±39	134±39	0,709^a^
HDL-C (mg/dL)	48±18	45±12	0,301^a^
CT (mg/dL)	204±48	203±43	0,880^a^
TG (mg/dL)	174±143	166±147	0,541^a^
DDFVE (mm)	46,1±4,4	47,9±4,6	0,037^a^
DSFVE (mm)	30,9±5,8	33,3±5,6	0,025^a^
SIV (mm) *	11 (8-14)	12 (9-15)	0,000^c^
PP (mm) *	11 (8-14)	11 (9-14)	0,045^c^
AE (mm) *	36 (24-47)	36 (30-57)	0,286^c^
FE (%)*	55 (35-65)	55 (25-65)	0,009^c^
DD (n, %)	31 (67)	62 (65)	0,669^b^

^a ^Teste t independente, ^b ^Teste do qui-quadrado, ^c ^Teste U de Mann–Whitney, * Os dados são expressos como mediana (intervalo interquartil) para as variáveis contínuas. AE: átrio esquerdo; ALT: alanina aminotransferase; AST: aspartato aminotransferase; CT: colesterol total; DD: disfunção diastólica; DDFVE: diâmetro diastólico final do ventrículo esquerdo; DSFVE: diâmetro sistólico final do ventrículo esquerdo; eTFG: taxa de filtração glomerular estimada; FE: fração de ejeção; HDL-C: colesterol de lipoproteína de alta densidade; LDL-C: colesterol de lipoproteína de baixa densidade; PCR: proteína C reativa; PP: parede posterior; SIV: septo interventricular; TG: triglicerídeos.

Os pacientes foram agrupados de acordo com tercis do escore SYNTAX mediano definidos da forma seguinte: pontuação SYNTAX baixa < 8 (n = 61, 43%) e pontuação SX alta ≥ 8 (n = 80, 57%). O número de pacientes com escores altos foi significativamente maior no grupo de hipertensão não-dipper em comparação com o grupo de hipertensão dipper (Tabela 1). Em um modelo de regressão logística multivariável, o status de hipertensão não-dipper surgiu como um preditor independente de alto escore SYNTAX. Outros preditores independentes de escore SYNTAX elevado incluíram sexo e colesterol LDL (Tabela 3).

**Tabela 3:** – Análise multivariada mostrando a associação entre os parâmetros e o escore SYNTAX

Variáveis	β	SE	Wald	OR (95% CI)	p
Idade	-0,016	0,024	0,424	0,984 (0,939-1,032)	0,515
Sexo	1,406	0,562	6,260	4,081 (1,356-12,282)	0,012
IMC	-0,051	0,054	0,903	0,950 (0,855-1,056)	0,342
HT	0,000	0,445	0,000	1,000 (0,418-2,389)	0,999
DM	-0,030	0,453	0,004	0,970 (0,399-2,357)	0,947
Tabagismo	0,056	0,456	0,015	1,058 (0,433-2,586)	0,901
eGFR	-0,013	0,014	0,856	0,987 (0,960-1,015)	0,355
LDL-C	0,015	0,005	7,048	1,015 (1,004-1,026)	0,008
HT não-dipper	1,641	0,424	14,952	5,159 (2,246-11,852)	0,000

*DM: diabetes mellitus; eGFR: taxa de filtração glomerular estimada; HT: hipertensão; IMC: índice de massa corporal; LDL-C: colesterol de lipoproteína de baixa densidade.*

## Discussão

Os nossos resultados sugeriram uma associação significativa entre a menor redução da PA sistólica noturna (hipertensão não-dipper) na MCPA frequente e a gravidade e complexidade da DAC calculada pelo escore SYNTAX em pacientes hospitalizados por SCA. Além disso, verificamos que a hipertensão não-dipper foi um indicador independente de escore SYNTAX mais alto nessas populações de pacientes.

Pela primeira vez, O’Brien et al.^19^ relataram que menor redução da PA noturna estava associada a maior prevalência de acidente vascular cerebral. Descreveram essa anormalidade circadiana da PA como hipertensão não-dipper. Desde então, um número crescente de relatos têm publicado que existe uma estreita relação entre hipertensão não-dipper e aumento da morbimortalidade cardiovascular.^2-7^ Em uma metanálise recente, o estudo ABC-H^7^ avaliou o seguimento de 8 anos de 17.312 pacientes com hipertensão. O padrão não-dipping, após ajuste para PA sistólica de 24 horas, foi um preditor de excesso de risco variando de 33% para mortalidade por todas as causas a 57% para mortalidade cardiovascular. Mousa et al.^20^ demonstraram uma associação significativa entre hipertensão não-dipper e DAC significativa (≥ 70% de estenose de artéria coronária na angiografia) independente de outros parâmetros clínicos em homens. Wirtwein et al.^21^ relataram que a extensão da estenose significativa da artéria coronária (≥ 50% de estenose em pelo menos três artérias coronárias na angiografia) e eventos cardiovasculares adversos maiores estavam relacionados a dipping reduzido da PA sistólica noturna.

Embora a estreita correlação entre hipertensão não-dipper e eventos cardiovasculares adversos maiores tenha sido demonstrada em muitos estudos diferentes, o mecanismo fisiopatológico subjacente permanece incerto. Foi comprovado que o escore SYNTAX reflete eventos cardiovasculares adversos maiores, como na hipertensão não-dipper. Portanto, os resultados do nosso estudo podem fornecer evidências adicionais para tal correlação ao revelar DAC mais intensa e mais complexa calculada pelo escore SYNTAX em pacientes com SCA e hipertensão não-dipper. Entre os mecanismos mais enfatizados na patogênese estão a mudança para hiperatividade simpática no sistema nervoso autônomo à noite, disfunção dos barorreceptores arteriais, labilidade elevada de repolarização miocárdica, aumento da sensibilidade ao sódio, aumento da rigidez arterial, inflamação crônica de baixo grau e disfunção endotelial.^22-27^ A diminuição do tônus arterial à noite faz com que a PA pulsátil seja transmitida à microcirculação de forma mais eficaz do que durante o dia e interrompe o fluxo laminar fisiológico. Como resultado, as células endoteliais arteriais são expostas a estresse de cisalhamento oscilante; a biodisponibilidade do óxido nítrico diminui; o estresse oxidativo aumenta e a disfunção endotelial, que é o primeiro passo no desenvolvimento da aterosclerose, é estimulada.^28,29^ As lesões coronarianas complexas avaliadas pelo escore SYNTAX, como ramos, bifurcações e curvaturas, estão intimamente relacionadas ao estresse de cisalhamento oscilante, apoiando essa hipótese.^30^ Demonstramos anteriormente que, em pacientes com SCA, os marcadores de estresse oxidativo aumentaram significativamente na DAC intensiva avaliada pelo escore de Gensini.^31^ Além disso, mostrou-se que precursores de trombogênese como fator de von Willebrand, D-dímero, fibrinogênio e P-selectina são significativamente aumentados em pacientes com hipertensão não-dipper e DAC, apoiando o mecanismo associado à SCA.^32^ Também encontramos uma porcentagem maior de IAMCSST em pacientes com hipertensão não-dipper, nos quais a trombose é mais proeminente na sua fisiopatologia do que em outros tipos de SCA.^33^

A MAPA é considerada o padrão ouro para monitorização da PA noturna; no entanto, métodos alternativos começaram a se desenvolver, principalmente em pacientes hospitalizados, devido ao uso clínico limitado da MAPA, seu alto custo e o fato de interferir no conforto do sono. Xu et al.^8^ mediram, com esfigmomanômetro manual, 6 vezes ao dia, em intervalos de 4 horas em pacientes hospitalizados, os valores que denominaram MCPA em comparação com a MAPA tradicional de 24 horas. Os pesquisadores relataram uma forte correlação entre a PA clínica e ambulatorial para a PA sistólica e diastólica. Além disso, declararam que a detecção de não-dipper pela MCPA estava bem de acordo com a MAPA de 24 horas. Além disso, desde que um monitor automático de pressão arterial domiciliar foi desenvolvido pela primeira vez em 2001 e usado para monitorazação noturno da PA em um estudo, muitos estudos confirmaram uma forte correlação entre as medidas de MAPA e MDPA. Tem sido relatado na literatura que a MDPA pode ser uma alternativa confiável à MAPA para avaliar a PA noturna e detectar hipertensão não-dipper.^9,10,34^ Recentemente, os dados do estudo J-HOP Nocturnal BP, a maior coorte de MDPA baseada na prática, mostraram que um aumento de 10 mmHg na PA sistólica noturna, na MDPA, foi associado a um aumento significativo de 20,1% nos eventos cardiovasculares adversos maiores, semelhantes àqueles medidos pela MAPA.^35^ Embora Xu et al.^8^ tenham calculado MCPA em pacientes hospitalizados, realizando 3 medições de PA diurnas e 3 noturnas em um único dia, na maioria dos estudos com MDPA, 3 medições de PA diurnas e 3 noturnas foram repetidas ao longo de 1 a 2 semanas e foram calculadas as médias. Para superar a permanência limitada na unidade de terapia intensiva intermediária e a falta de oportunidade de medir em dias repetidos sob as mesmas condições, realizamos a MCPA em intervalos frequentes (uma vez por hora) como uma combinação de ambos esses métodos no nosso estudo.

### Limitações do estudo

O nosso estudo possui algumas limitações:

A reprodutibilidade não pôde ser analisada, pois foi possível medir a PA em apenas um dia. Para superar esse problema, usamos a MCPA frequente, um protocolo modificado de MCPA, no nosso estudo.

Embora tenhamos prestado o máximo de atenção para garantir as condições e níveis de sono-vigília ideais, a qualidade do sono que poderia afetar a PA noturna não foi avaliada no nosso estudo.

Mesmo com os dois grupos comparados estando nas mesmas condições, o período de hospitalização imediatamente após a SCA pode afetar o estado de estresse e o sistema nervoso autônomo dos pacientes, causando resultados diferentes da condição estável.

## Conclusão

Os resultados do nosso estudo revelaram a relação entre o escore SYNTAX e a hipertensão não-dipper em pacientes com SCA, até onde sabemos pela primeira vez, fornecendo um possível mecanismo adicional ligando a PA circadiana anormal a doenças cardiovasculares. Estudos futuros são necessários para melhor compreensão dessa associação e determinar as abordagens necessárias para a PA diurna ideal. Duração mais longa e múltiplas múltiplas medições pressóricas de 24 horas podem ser mais informativas a esse respeito.
