# Supplementary material for: A Variação Anormal da Pressão Arterial Circadiana está Associada aos Escores SYNTAX em Pacientes Hospitalizados com Síndrome Coronariana Aguda
Source: Arq Bras Cardiol. 2022 Apr 26;119(1):76–84. [Article in Portuguese] doi: 10.36660/abc.20210546 (PMC9352112; doi:10.36660/abc.20210546)
Supplement: Translation

# Translation: Abnormal Circadian Blood Pressure Variation is Associated with SYNTAX Scores in Hospitalized Patients with Acute Coronary Syndrome

**Authors:** Turhan Turan, Ahmet Özderya, Sinan Sahin, Selim Kul, Ali Hakan Konuş, Faruk Kara, Gulay Uzun, Ali Rıza Akyüz, Muhammet Rasit Sayin

**Affiliations:** 1 Turkey Trabzon Ahi Evren Thoracic and Cardiovascular Surgery Training and Research Hospital - University of Health Sciences, Trabzon – Turkey

**Keywords:** Hypertension, Blood Pressure Monitoring Ambulatory, Acute Coronary Syndrome, Inpatients, Coronary Artery Disease

## Abstract

**Background:**

Blunted nocturnal blood pressure (BP) reduction, referred to as non-dipper hypertension, is a strong predictor of cardiovascular morbidity and mortality.

**Objectives:**

This study aimed to investigate the relationship between non-dipper hypertension and the severity and complexity of coronary artery disease using SYNTAX score in hospitalized patients with acute coronary syndrome.

**Methods:**

A total of 306 consecutive patients with acute coronary syndrome were screened. Patients who were clinically stable and admitted to the intermediate intensive care unit at least 24 hours after angiography and/or successful revascularization. After the exclusion criteria, 141 patients (34 female and 107 male; mean age 61 ± 11 years) were included. Non-dipper hypertension has been defined as a 0% to 10% decrease in average systolic BP at nighttime compared to daytime, measured at hourly intervals using the same automatic BP measuring device on bedside monitors (Vismo PVM-2701; Nihon Kohden Corp., Tokyo, Japan). SYNTAX score was calculated with an online calculator. The independent predictors of SYNTAX score were assessed using multivariable logistic regression analysis. P < 0.05 was considered statistically significant.

**Results:**

The patients with non-dipper hypertension had higher SYNTAX score than the patients with dipper hypertension (11.12 ± 6.41 versus 6.74 ± 6.45, p < 0.0001). In a multivariable logistic regression model, non-dipper hypertension status (odds ratio: 5.159; 95% confidence interval: 2.246 to 11.852, p < 0.001), sex (p = 0.012) and low-density lipoprotein cholesterol (p = 0.008) emerged as independent predictors of high SYNTAX score.

**Conclusions:**

The results of our study provide a possible additional mechanism linking abnormal circadian BP profile with coronary artery disease severity and complexity in patients with acute coronary syndrome.

## Introduction

Physiological blood pressure (BP) exhibits a circadian pattern with a decrease of 10% to 20% during sleep in relation to daytime BP. This decrease during sleep is defined as extreme dipping if ≥ 20%, normal dipping if 10% to 20%, non-dipping if < 10%, and reverse dipping if there is any increase (night to day ratio: ≤ 0.8, < 0.8 to ≤ 0.9, < 0.9 to ≤ 1, and > 1, respectively).^1^ Blunted nocturnal BP reduction is a strong predictor of cardiovascular morbidity and mortality for patients with and without hypertension.^2-7^ The standard method for determining non-dipper and dipper patterns in patients is 24-hour non-invasive ambulatory blood pressure monitoring (ABPM), which is generally performed out of the office. On the other hand, alternatively, clinical blood pressure monitoring (CBPM) in hospitalized patients and home blood pressure monitoring (HBPM) in outpatients are performed with infrequent manual or automatic BP measurements. CBPM and HBPM have previously shown to have daytime and nighttime BP measurements similar to ABPM in hospitalized patients and outpatients and to be consistent with ABPM in demonstrating non-dipper hypertension.^8-10^

Although diurnal BP disorders are linked to damage of several organs and cardiovascular events, the underlying mechanism is unclear.^11-13^ However, the clinical significance of abnormal circadian BP variation in patients admitted to the hospital with a recent cardiovascular event has not yet been studied. The Synergy between Percutaneous Coronary Intervention with Taxus and Cardiac Surgery Study (SYNTAX) score (SX score) is one of the most accepted detailed coronary angiographic scoring systems for determining the severity and complexity of coronary artery disease (CAD), depending on coronary anatomy and lesion characteristics.^14-16^ This study aimed to evaluate the relationship between SX score and blunted nighttime BP dipping using frequent CBPM (hourly intervals) in hospitalized patients with acute coronary syndrome (ACS).

## Methods

### Study population

This single-center, cross-sectional, prospective study was held between January and April 2020 at the Ahi Evren Thoracic and Cardiovascular Centre, Trabzon, Turkey. The study participants were prospectively enrolled from a total of 306 patients who had undergone coronary angiography with ACS (ST-elevation myocardial infarction [STEMI], non-STEMI [NSTEMI], unstable angina pectoris [USAP]). Biochemistry parameters, including total cholesterol, low-density lipoprotein (LDL), high-density lipoprotein (HDL), triglycerides, and renal function tests, were measured. Hematological parameters were measured as a part of the automated complete blood count (Mindray BC-5800 auto hematology analyzer, Mindray Medical Electronics Co. Shenzhen, China). Hypertension was diagnosed and stratified based on recent guidelines.^17^ Patients who had previously received antihypertensive treatment continued the same treatments throughout the follow-up period. We gave antihypertensive medicines to all patients in the morning hours without changing their usage. Hypercholesterolemia was defined as total cholesterol > 200 mg/dl. The estimated glomerular filtration rate was calculated using the Cockcroft-Gault formula.^18^ We excluded patients with any of the following: presenting with cardiogenic shock or arrest, receiving intravenous nitroglycerine or inotrope therapy for any reason, history of coronary artery bypass grafting, valvular heart disease, malignancy, renal or hepatic disease, symptomatic heart failure, secondary hypertension, uncontrolled arrhythmia, ongoing angina or anxiety, obstructive sleep apnea syndrome or sleep disorder, and morbid obesity (body mass index > 35). Finally, the study population consisted of 141 clinically stable patients, including 85 with NSTEMI, 15 with USAP, and 41 with STEMI (Figure 1). Patient ages ranged from 32 to 91 years. The study protocol conformed to the principles of the Declaration of Helsinki and received approval from the local Institutional Review Board. Informed consent was obtained from each study participant.

**Figure 1:** – Flowchart of the study. ACS: acute coronary syndrome.

### Coronary angiography

All patients underwent coronary angiography within 24 hours. The average time from symptoms to coronary angiography was approximately 2 to 6 hours. Coronary angiography was performed by the standard Judkins technique using 6 or 7 French catheters (Expo, Boston Scientific Corporation, Massachusetts, USA) through the femoral artery. When necessary, percutaneous coronary intervention for the culprit lesion was successfully performed on eligible patients in the same session (120/141 patients, 85%). SX score was calculated with an online calculator as described in the literature according to the basal angiographic findings by 2 experienced operators who were blind to other parameters.^16^

### Blood pressure measurement and study protocol

In our clinic, patients with ACS are followed up in the intensive care unit for at least the first 24 hours after percutaneous coronary intervention. However, low-risk stable patients (patients with successful revascularization, no malignant arrhythmia, relieved pain, and no signs of heart failure) are mobilized and followed up in the intermediate intensive care unit at the end of 24 hours. Our study population was selected from these patients, and hourly BP measurements were made in the intermediate intensive care unit with an automatic BP measurement device on bedside monitors for all patients (Vismo PVM-2701, Nihon Kohden Corp., Tokyo, Japan). Measurements were made in the upper limb using 2 inflatable cuffs (22 × 12 and 30 × 14 cm) to cover at least 80% of the patient’s arm circumference. Measurement accuracy was ensured by optimizing all patient monitors before the first measurements and comparing them with calibrated standard sphygmomanometer measurements. By enabling the patients to sleep in their beds at 23:00 and wake up at 7:00, nighttime BP values were obtained by hourly measurement. We informed all patients before the procedure and took all nighttime BP measurements while the patients were sleeping.

We excluded measurement if the patient awoke for any reason, and the measurement was repeated immediately after the patient slept. Coffee drinking, smoking, and exercise were not allowed before measurement; after sitting and resting for 5 minutes, daytime BP was obtained by measuring BP hourly between 08:00 and 22:00 with the same device in the supine position. For all measurements, patient monitors were set to measure at 1-hour intervals. The same experienced healthcare personnel checked the patients in terms of sleep-wake status, cuff appropriateness, and patient position during measurement; nighttime BP measurements were recorded under dim light, without fully turning on the intermediate intensive care unit lights. If the patient was transferred to the intermediate intensive care unit at a time that did not correspond to the beginning of the measurement periods, we started the measurements in the first following period (23:00 at night-time or 7:00 the daytime). Patients were followed up in the intermediate intensive care unit for at least 24 hours. Extreme BP values (systolic BP > 200 mmHg or < 90 mmHg; diastolic BP > 110 mmHg or < 40 mmHg) were considered as erroneous measurement and were not included in the analysis. By averaging the hourly BP values for 9 nighttime periods (23:00 to 07:00) and 15 daytime periods (8:00 to 22:00), a single daytime and nighttime mean BP value was obtained. Nighttime to daytime systolic BP dip was calculated as 100 × ([day-time systolic BP mean – nighttime systolic BP mean]/daytime systolic BP mean]). Decrease in BP at nighttime compared to daytime was defined in the following manner: normal dipping if 10% to 20%, non-dipping if < 10%, and reverse dipping if there was any increase (night to day ratio: ≤ 0.8, < 0.8 to ≤ 0.9, < 0.9 to ≤ 1, and > 1, respectively).

### Echocardiographic evaluation

Echocardiographic examination was performed with a commercially available cardiovascular ultrasound system (Vivid 5, GE Vingmed, Horten, Norway). Data acquisition was performed with a 1.5 to 2.6 MHz transducer in the parasternal and apical views (standard 2- and 4-chamber views). Two-dimensional and Doppler images were obtained during breath-hold and stored in cine-loop format from 3 consecutive beats; average values were reported, and electrocardiograms were simultaneously recorded. Left ventricle ejection fraction was derived by the apical biplane modified Simpson rule. Doppler measurements included peak early mitral filling velocity (E wave), peak late mitral filling velocity (A wave), and the ratio of peak early and late mitral filling velocities (E/A). For myocardial tissue velocities, tissue Doppler imaging sample volume was placed at the lateral mitral annulus at the junction between the left ventricular lateral wall and mitral annulus apical 4-chamber view. Tissue Doppler imaging included the following parameters: early diastolic myocardial velocity (Em), late diastolic myocardial velocity (Am), and Em/Am. All echocardiograms were interpreted by two experienced cardiologists who were blind to patient status.

### Statistical analysis

SPSS 21.0 for Windows (SPSS Inc., Chicago, IL, USA) was used for statistical analyses. Quantitative data were expressed as mean ± standard deviation. Categorical data were presented as number and frequency (%). For the suitable analysis technique, Kolmogorov–Smirnov and homogeneity of variance tests were applied. Independent sample t-tests were used for two-group comparison of the normally distributed variables, and Mann–Whitney U-test was used for two-group comparison of the variables without normal distribution. Non-normally distributed variables were expressed as medians (interquartile ranges). Normally distributed continuous variables were expressed as mean ± standard deviation. Categorical variables were compared using the chi-square test. Independent predictors of SX score were assessed using multivariable logistic regression analysis. In the multivariate procedure, age, sex, body mass index, history of hypertension and diabetes mellitus, smoking, glomerular filtration rate, LDL cholesterol levels, and non-dipper hypertension were the clinical variables considered. P < 0.05 was considered statistically significant.

## Results

In this study, a total of 306 consecutive patients with ACS were screened. After excluding patients who met the exclusion criteria, the remaining 141 patients (34 female and 107 male; mean age 61 ± 11 years) were included in the study (Figure 1). STEMI, NSTEMI, and USAP were observed in 41 (29%), 85 (60%), and 15 (11%) patients, respectively. Among all patients with ACS, non-dipper hypertension was observed in 95 (67%) patients. The clinical characteristics of patients are displayed in Table 1. There were noticeable significant clinical differences between the groups. Patients with non-dipper hypertension had higher STEMI and lower USAP percentage than patients with dipper hypertension. Patients with non-dipper hypertension also had higher SX score, higher peak high-sensitivity troponin I levels, higher left ventricular dimensions, and lower ejection fraction than the patients with dipper hypertension (Tables 1 and 2 and Figure 2).

**Table 1:** – Baseline clinical characteristics of the study population

	Dipper group (n=46)	Non-dipper group (n=95)	p
Age (years)	61±12	61±11	0.868^a^
Male sex (n) (%)	35 (76)	72 (76)	0.969^a^
BMI (kg/m^2^)	27.4±3.8	28.0±3.7	0.395^a^
HT (n, %)	23 (50)	48 (51)	0.953^b^
DM (n, %)	12 (26)	29 (31)	0.586^b^
Smoking (n, %)	14 (30)	36 (38)	0.385^b^
Mean daytime BP (mmHg, systolic/diastolic)	121.3±13.9/ 73.4±9.2	118.2±15.4/ 69.5±10.3	0.251^a^ 0.035^a^
Mean nighttime BP (mmHg, systolic/diastolic)	103.5±12.3/ 63.0±8.2	118.2±15.4/ 70.5±9.8	<0.001^a^ <0.001^a^
ACS type:			
STEMI (n, %)	7 (15)	34 (36)	0.012^b^
NSTEMI (n, %)	29 (63)	56 (59)	0.641^b^
USAP (n, %)	10 (22)	5 (5)	0.030^b^
SYNTAX Score*	5 (0-21)	9.5 (0-29)	<0.00^1^c
High SYNTAX Score (n, %)^ #^	16 (35)	64 (67)	<0.001^b^
Medications			
ACE inhibitor or ARB (n, %)	16 (35)	30 (32)	0.704^b^
Calcium antagonist (n, %)	6 (13)	21 (22)	0.200^b^
Diuretics (n, %)	8 (17)	17 (18)	0.942^b^
MRA (n, %)	1 (2)	2 (2)	0.979^b^
Β-blocker (n, %)	10 (22)	27 (28)	0.398^b^
α-blocker (n, %)	1 (2)	0 (0)	0.149^b^
Clopidogrel	35(76.1)	84(88)	0.082^ b^
Ticagrelol	8 (17)	9 (9.5)	0.180^ b^
Prasugrel	3 (6.5)	2(2.1)	0.330^ b^
Acetylsalicylic acid	46 (100)	95 (100)	1^ b^
Statin	46 (100)	93 (98)	1^ b^

^a ^Independent t test, ^b ^Chi-square test, ^c ^Mann–Whitney U test, * Data are expressed as median (interquartile range) for continuous variables. ^#^Above the median value. ACE: angiotensin converting enzyme; ACS: acute coronary syndrome; ARB: angiotensin receptor blocker; BMI: body mass index; BP: blood pressure; DM: diabetes mellitus; HT: hypertension; MRA: mineralocorticoid receptor antagonist; NSTEMI: non-ST-elevation myocardial infarction; STEMI: ST-elevation myocardial infarction; USAP: unstable angina pectoris.

**Table 2:** – Biochemical values and echocardiographic parameters of the study population

	Dipper group n=46	Non-dipper group n=95	p
Peak hs-troponin I (ng/L) *	2413 (13.9-50000)	9036 (1.01-150050)	0.021^c^
Creatinine (mg/dL) *	0.94 (0.56-1.66)	0.87 (0.63-1.86)	0.361^c^
eGFR (ml/dk/1,73 m^2^) *	83.5 (34-114)	89.6 (32.9-118)	0.737^c^
Sodium (mmol/L)	137±2	137±2	0.549^a^
Potassium (mmol/L)	4.3±0.3	4.3±0.4	0.930^a^
Calcium (mg/dL)	9.0±0.4	8.8±0.5	0.058^a^
ALT (IU/L) *	24 (9-94)	24 (6-150)	0.418^c^
AST (IU/L) *	35.5 (15-291)	38 (10-472)	0.809^c^
CRP (mg/L) *	2.65 (0-79)	2.4 (0-93)	0.974^c^
FBG (mg/dL) *	122.5 (74-367)	127 (59-316)	0.427^c^
LDL-C (mg/dL)	137±39	134±39	0.709^a^
HDL-C (mg/dL)	48±18	45±12	0.301^a^
TC (mg/dL)	204±48	203±43	0.880^a^
TG (mg/dL)	174±143	166±147	0.541^a^
LV-EDD (mm)	46.1±4.4	47.9±4.6	0.037^a^
LV-ESD (mm)	30.9±5.8	33.3±5.6	0.025^a^
IVS (mm) *	11 (8-14)	12 (9-15)	0.000^c^
PW (mm) *	11 (8-14)	11 (9-14)	0.045^c^
LA (mm) *	36 (24-47)	36 (30-57)	0.286^c^
EF (%)*	55 (35-65)	55 (25-65)	0.009^c^
DD (n, %)	31 (67)	62 (65)	0.669^b^

^a ^Independent t test,^ b ^Chi-square test, ^c ^Mann–Whitney U test * Data are expressed as median (interquartile range) for continuous variables. ALT: alanine aminotransferase; AST: aspartate aminotransferase; CRP: C-reactive protein; DD: diastolic dysfunction; EF: ejection fraction; eGFR: estimated glomerular filtration rate; FBG: fasting blood glucose; HDL-C: high-density lipoprotein cholesterol; hs-troponin I: high-sensitivity troponin I; IVS: interventricular septum; LA: left atrium; LDL-C: low-density lipoprotein cholesterol; LV-EDD: left ventricular end-diastolic diameter; LV-ESD: left ventricular end-systolic diameter; PW: posterior wall; TC: total cholesterol; TG: triglycerides.

**Figure 2:** – Comparison of SYNTAX scores of patients in dipper and non-dipper hypertension groups (11.12 ± 6.41 versus 6.74 ± 6.45, p < 0.001).

HT: hypertension.

Patients were grouped according to median SX score tertiles defined as: low SX score < 8 (n = 61, 43%) and high SX score ≥ 8 (n = 80, 57%). The number of patients with high scores was significantly higher in the non-dipper hypertension group compared to the dipper hypertension group (Table 1). In a multivariable logistic regression model, non-dipper hypertension status emerged as and independent predictor of high SX score. Other independent predictors of high SX score included sex and LDL cholesterol (Table 3).

**Table 3:** – Multivariate analysis showing the association between parameters and Syntax score

Variables	β	SE	Wald	OR (95% CI)	p
Age	-0.016	0.024	0.424	0.984 (0.939-1.032)	0.515
Sex	1.406	0.562	6.260	4.081 (1.356-12.282)	0.012
BMI	-0.051	0.054	0.903	0.950 (0.855-1.056)	0.342
HT	0.000	0.445	0.000	1.000 (0.418-2.389)	0.999
DM	-0,030	0.453	0.004	0.970 (0.399-2.357)	0.947
Smoking	0.056	0.456	0.015	1.058 (0.433-2.586)	0.901
eGFR	-0.013	0.014	0.856	0.987 (0.960-1.015)	0.355
LDL-C	0.015	0.005	7.048	1.015 (1.004-1.026)	0.008
Non-dipper HT	1.641	0.424	14.952	5.159 (2.246-11.852)	0.000

*BMI: body mass index; DM: diabetes mellitus; eGFR: estimated glomerular filtration rate; HT: hypertension; LDL-C: low-density lipoprotein cholesterol.*

## Discussion

Our results suggested a significant association between blunted nocturnal systolic BP reduction (non-dipper hypertension) on frequent CBPM and the severity and complexity of CAD calculated by SX score among patients hospitalized for ACS. Also, we found that non-dipper hypertension was an independent indicator of higher SX score in these patient populations.

For the first time, O’Brien et al.^19^ reported that the blunted decrease in nighttime BP was associated with a higher prevalence of stroke. They described this circadian BP abnormality as non-dipper hypertension. Since then, a growing number of reports have published that there is a close relationship between non-dipper hypertension and increased cardiovascular morbidity and mortality.^2-7^ In a recent meta-analysis, the ABC-H^7^ study evaluated the 8-year follow-up of 17,312 hypertension patients. The non-dipping pattern, after adjustment for 24-hour systolic BP, predicted an excess risk ranging from 33% for all-cause mortality to 57% for cardiovascular mortality. Mousa et al.^20^ showed a significant association between non-dipper hypertension and significant CAD (≥ 70% coronary artery stenosis on angiography) independent of other clinical parameters in men. Wirtwein et al.^21^ reported that extent of significant coronary artery stenosis (≥ 50% stenosis in at least three coronary arteries on angiography) and major adverse cardiovascular events were related to blunted nighttime systolic BP dipping.

Although the close correlation between non-dipper hypertension and major adverse cardiovascular events has been demonstrated in many different studies, the underlying pathophysiological mechanism remains unclear. SX score has been proven to reflect major adverse cardiovascular events as in non-dipper hypertension. Therefore, the results of our study may provide additional evidence for such correlation by revealing more intense and more complex CAD calculated by SX score in patients with ACS and non-dipper hypertension. Among the most emphasized mechanisms in pathogenesis are the shift to sympathetic overactivity in the autonomic nervous system at night, arterial baroreceptor dysfunction, elevated myocardial repolarization lability, increased sodium sensitivity, increased arterial stiffness, chronic low-grade inflammation, and endothelial dysfunction.^22-27^ Decreased arterial tone at night causes pulsatile BP to be transmitted to microcirculation more effectively than during the day and disrupts the physiological laminar flow. As a result, arterial endothelial cells are exposed to oscillatory shear stress; nitric oxide bioavailability decreases; oxidative stress increases, and endothelial dysfunction, which is the first step in the development of atherosclerosis, is stimulated.^28,29^ Complex coronary lesions assessed by SX score, such as branches, bifurcations, and curvatures, are closely related to oscillating shear stress, supporting this hypothesis.^30^ We have previously shown that, in patients with ACS, oxidative stress markers significantly increased in intensive CAD assessed by the Gensini score.^31^ Moreover, precursors of thrombogenesis such as von Willebrand factor, D-dimer, fibrinogen, and P-selectin have been shown to be significantly increased in patients with non-dipper hypertension and CAD, supporting the mechanism associated with ACS.^32^ We also found a higher percentage of STEMI in patients with non-dipper hypertension, in which thrombosis is more prominent in its pathophysiology than in other ACS types.^33^

ABPM is considered the gold standard for monitoring nighttime BP; however, alternative methods have begun to develop, especially in hospitalized patients, due to the limited clinical use of ABPM, its high cost, and the fact that it disrupts sleep comfort. Xu et al.^8^ measured, with a manual sphygmomanometer, 6 times a day at 4-hour intervals in hospitalized patients the values they called CBPM compared with traditional 24-hour ABPM. The investigators reported a strong correlation between clinical and ambulatory BP for both systolic and diastolic BP. Also, they declared that the detection of non-dippers by CBPM was in good agreement with 24-hour ABPM. Moreover, since an automatic home blood pressure monitor was first developed in 2001 and used for nighttime BP monitoring in a study, many studies have confirmed a strong correlation between APBM and HBPM measurements. It has been reported in the literature that HBPM can be a reliable alternative to ABPM to evaluate nighttime BP and detect non-dipper hypertension.^9,10,34^ Recently, the J-HOP Nocturnal BP Study data, the largest practice-based HBPM cohort, showed that a 10 mmHg increase in nighttime systolic BP in HBPM was associated with a significant 20.1% increase in major adverse cardiovascular events, similar to those measured by ABPM.^35^ Although Xu et al.^8^ calculated CBPM in hospitalized patients by taking 3 daytime and 3 night-time BP measurements in a single day, in most HBPM studies, 3 daytime and 3 night-time BP measurements were repeated over 1 to 2 weeks and averaged. To overcome limited stay in the intermediate intensive care unit and the lack of opportunity to measure on repeated days under the same conditions, we performed CBPM at frequent intervals (once an hour) as a combination of both of these methods in our study.

### Study limitations

Our study has some limitations:

Reproducibility could not be analyzed, as it was possible to measure BP for only one day. To overcome this problem, we used frequent CBPM, a modified CBPM, in our study.

Although we paid the maximum attention to ensure optimal conditions and sleep-wake levels, sleep quality that could affect nighttime BP was not evaluated in our study.

Even if both groups compared were in the same conditions, the hospitalization period immediately after ACS might affect the patients’ stress state and autonomic nervous system, causing different results from the stable condition.

## Conclusion

Our study results revealed the relationship between SX score and non-dipper hypertension in patients with ACS, to the best of our knowledge for the first time, providing a possible additional mechanism linking abnormal circadian BP with cardiovascular diseases. Further studies are needed to clarify this association and determine the approaches required for optimal diurnal BP. Longer duration and multiple 24-hour BP measurements may be more informative in this regard.
